# Functioning of Heat Accumulating Composites of Carbon Recyclate and Phase Change Material

**DOI:** 10.3390/ma15062331

**Published:** 2022-03-21

**Authors:** Michał Musiał, Agnieszka Pękala

**Affiliations:** 1 Department of Building Engineering, Rzeszów University of Technology, ul. Poznańska 2, 35-959 Rzeszów, Poland; 2 Department of Environmental Engineering and Chemistry, Rzeszów University of Technology, al. Powstanców Warszawy 6, 35-959 Rzeszów, Poland; apekala@prz.edu.pl

**Keywords:** composite phase change material, energy-saving construction, coke recycling, heat storage, biochar, closed cycle, pyrolysis, coke

## Abstract

The article presents the results of experimental research together with the development of a response function presenting the thermal functioning of a new composite of a phase change material with carbon recyclate. The empirical research proved the improvement of the thermal functioning of the phase change material as a result of modifying its structure with carbon-based recycling material. The conducted experimental tests and statistical analysis proved that the obtained innovative composite is characterized by a more effective distribution of stored heat than the pure phase change material, which resulted in reduction of the heating and cooling time of the package by 10 min. The obtained innovative composite can improve the thermal efficiency of short-term heat storage systems, both in building components and in elements of heating and cooling systems, and translates into their increase in thermal efficiency.

## 1. Introduction

The issues discussed in the article present an innovative approach to the management of industrial waste as components of phase change materials. So far, there have been no studies in which the matrix of the phase change composite was created on the basis of carbonate recyclates. The conducted experiment signs in both the chain of waste management activities, consistent with the idea of a circular economy, and the trend of using new, energy-saving solutions in construction. The search for new solutions in construction results from the need to reduce the energy demand of buildings and the gases they emit. The increasing demand for electricity and heat in the world forces an increase in the amount of energy obtained from renewable sources.

The main source of renewable energy for buildings is solar radiation energy. The limitations of obtaining heat for the heating purposes of buildings from this renewable source are: weather conditions changing with time, the nature of the external climate, and the occurrence of changing times of the day and seasons. For this reason, the gains from solar radiation are characterized by high instantaneous efficiency and, at the same time, high variability of the acquisition of thermal energy.

One of the possibilities of more effective use of gains from solar radiation for heating buildings are passive solar systems based on both direct and indirect gains from solar radiation. Passive solar systems focusing on short-term storage of solar radiation energy in their structure, according to [1], have been used in construction since antiquity. Passive solar systems include building components that store thermal energy: walls, ceilings, windows, elements of heating installations, glass outbuildings, winter gardens, and collector and accumulation partitions. Due to their appropriate application, according to the works [1,2], they reduce daily temperature fluctuations of the thermal envelope of the building and its internal air thanks to the short-term storage of the absorbed solar radiation energy. According to work [3], thermal energy storage in building components and in its installed systems can be intensified through the use of phase-change materials (PCM). Phase change materials, according to the works [2,4,5], are characterized by isothermal absorption, storage, and release of thermal energy in the temperature range of phase transformations. In cooperation with building components, according to the works [4,5,6,7,8], phase change materials are mainly used with melting/freezing points in the range of 18–30 °C, where they have the highest possible enthalpy of phase change. According to the works [3,4,5], in construction, organic compounds or their mixtures (saturated hydrocarbons, alcohols, carboxylic acids, esters, and some polymers) are most commonly used as PCM..

The use of the above groups of organic phase change materials in construction is due to: repeatability of phase transformations of the material, chemical indifference to neighboring building materials, low price, and easy access to this group of materials. Nevertheless, for the wide use of this group of phase change materials in construction, it is necessary to eliminate or significantly reduce the main disadvantages of these materials according to [4,5,9], such as: flammability, low factor of organic thermal conductivity PCM in solid state, and ensuring tight warp for unused PCM packages.

The problem of increasing the efficiency of heat distribution within larger capsules and packets of PCM is key to the widespread use of PCM heat accumulators in construction due to the fact that the melting and solidification of PCM within a sealed package functioning as a building component takes place from the outside. According to [3,4,6,8,9], the thermal conductivity of solid PCMs in the group of organic compounds is in the range of 0.1–0.3 W/mK, which proves its heat-insulating nature. The high thermal resistance of organic PCM in the solid state reduces heat distribution during its heating and cooling. As a result, the full heat storage potential of the packets and larger PCM capsules is not fully utilized during the melting process. Likewise, during solidification, the change in aggregate state of the PCM takes place starting from the outer surfaces of the capsule, preventing the stored heat from being effectively released to the environment. According to the works [4,10,11,12,13,14,15,16,17], the improvement of the efficiency of heat distribution within PCM packages can be achieved by using in their structure a material that conducts heat well, such as copper [11,12,14], aluminum [13], or graphite [10].However, due to their high price and high carbon trace, the secant be replaced with other, more ecological substances at least in part from recycling.

This direction seems appropriate, in particular in terms of the innovative use of industrial waste from coal pyrolysis within PCM packages. The management of industrial waste and the consumption of technological products derived from coal pyrolysis is very important from an ecological point of view. In this context, it is justified to use easily available recycled materials, which can be used interchangeably as substances useful in other branches of the economy, to improve heat distribution within the PCM package.

The management of industrial waste and the consumption of technological products is very important from an ecological point of view. These issues are very complex and require planning and detailed chemical, technological, and experimental research. The article attempts to innovatively use solid raw material carbon recyclate residue from the pyrolysis process. Pyrolysis is the breakdown of molecules of complex chemical compounds into compounds of lower molecular weight under the influence of elevated temperature and in the absence of oxygen or other oxidizing agents. The pyrolysis of organic substances leads to a solid residue, the so-called biochar, and separation of volatile parts in the form of pyrolysis tar and pyrolysis gas. On an industrial scale, the aim of the pyrolysis of organic materials is the processing of raw materials (coal, biomass) into useful forms of energy, raw material recycling, and the production of semi-finished raw materials for their further use [18]. In highly developed countries, work is underway to improve and increase the efficiency of biomass combustion processes and the co-incineration of biomass with coal and in synthetic gas circuits. In addition to the development of technology, great emphasis is also placed on the search for new methods of the biomass processing of polymeric materials that have previously caused problems in processing [19,20]. Work is also conducted on the use of carbon biochar and waste rubber biochar as a pigment for black varnishes and for use in the production of steel sheets [21]. The use of pyrolytic waste carbon from tire combustion was demonstrated [22], and the use of coke resulting from the pyrolysis of sewage waste was the subject of research [23]. The rational utilitarian management of pyrolysis products allows the method of waste processing to become part of the chain of waste management activities consistent with the idea of a circular economy [24]. It is particularly important to define the possibility of a useful application of biochars obtained from the pyrolysis of various types of waste [25,26].Phase change materials are used in construction and other industries, creating new mixtures and composites and allowing for more effective heat storage in their structure. Below, in [27,28,29,30,31,32,33,34,35,36], examples of work on PCM composites are presented in order to improve their properties and increase the possibility of using the min construction.

The heat function of the new composite was presented in work [27], where the results of empirical research on the combination of an inorganic phase change material of calcium chlorine hexahydrate with hydrophilic colloidal silica were discussed. As a result, while ensuring the heat accumulation capacity of PCM, a composite was obtained that allows it to stabilize its form. The new composite was characterized by having a lower melting temperature of phase conversion than pure PCM by 7.81 °C and full phase conversion reversibility. The use of free PCM with the addition of pyrolytic carbon obtained from tire recycling is presented in work [28]. The composite obtained as a result of the research was used as an additive to concrete. The organic PCM RT21 used as an additive to concrete was added in a free state, and the recycling material made it possible to maintain it in the structure of the concrete element with a content of up to 32%. Article [29] presents the results of experimental studies on the PCM composite and the expanded glass aggregate, which acted as its carrier, with an adsorption coefficient of 80%. The obtained composite was characterized by high tightness, keeping the thermodynamic capabilities of PCM and significantly reducing the heat permeability coefficient of concrete by 47%. On the other hand, [30] presents an interesting method of soaking the matrix with liquid PCM. The tubular carbon fiber matrix of the composite was soaked with liquid PCM due to the use of centrifugal force during its rotation. The results of research on the composite of organic PCM in the form of stearic acid and ionic carbon fibers showed a yield point of 19 MPa and a phase change enthalpy of 77.83 J/g. An important aspect of using PCM as a heat accumulator is to improve the intensity of heat permeability, e.g., through the use of a conductive spatial structure. The authors in [13] presented a method of producing metal foams as a skeleton for metalfoam–PCM composites using Kelvin cells.

Another example of increasing the thermal conductivity of PCM described in work [31] is the composite of PCM and biochar obtained during the pyrolysis of invasive aquatic plants. The obtained composite was characterized by 13.82 times higher thermal conductivity than pure PCM. Phase change materials form cement-matrix composites that are used in construction as load-bearing components. The papers [32,33] present the results of strength tests of organic PCM composites with a slag aggregate matrix and silicon carbide, which confirmed its greater strength compared to the reference PCM composite with a matrix of natural aggregate. The properties of PCM that stabilize temperature changes are also used in the creation of food packaging. In work [34], the researchers conducted a study of thermal functioning on several composites of porous materials such as: bentonite clay, natural clay, diatomaceous earth, expanded perlite, activated carbon and silica gel with caprylic acid. Another example of the universal use of phase change materials is work [35], where the authors presented the PCM and nanographene composite as a component of clothing, having greater thermal accumulation and improving the thermal comfort of its owners.

The aspect of thermal energy storage is also important from the point of view of the construction of electric cars. Work [36] presents a heat storage system of phase change materials, allowing better management of thermal energy in electric cars.

The modeling of heat flows within phase change materials during their aggregate state transformation is possible by using finite difference method equations in the overt system [5,7,37] and finite element method equations [38]. On the other hand, in the case of experiments, the nature of which makes it impossible to perform many experiments, the determination of the influence of individual input quantities on the value of the output expression can be obtained without determining the form of the approximating function using artificial neural networks using the Mamdani–Assilian method, as in paper [39]. Determination of heat distribution within PCM elements—taking into account their geometry, heat flow intensity, and other input parameters—according to [40,41,42], is possible through the use of incomplete experimental plans, the selection of which for a specific object should take into account the criteria of: information, efficiency, and implementability. Carrying out the research cycle in accordance with an incomplete experiment plan allows for a significant reduction in the number of empirical experiments necessary from 125 to 17 and for obtaining the results of a statistical analysis of the function of the object.

The innovative contribution of this article is the development and testing of a new composite of an organic phase change material with carbon recyclate in the form of an internal skeleton of a heat accumulator. An approach different from that found in the scientific literature is the use of the proposed carbon recyclate in the new composite, instead of the expensive and necessary for other purposes copper alloys and aluminum. An additional utilitarian goal of this work is to obtain a mathematical dependence that allows us to determine the influence of: heat accumulator geometry, battery initial temperature, PCM heating temperature on the efficiency of heat distribution during melting, and solidification of the PCM contained in the heat accumulator.

Based on the literature review, the authors see the need to improve the efficiency of heat distribution within phase change heat accumulators and explore the possibility of obtaining it by applying it to the structure of organic PCM based on carbon recycle alkanes.

## 2. Materials and Methods

### 2.1. Materials and Apparatus Used in the Research

#### 2.1.1. Materials

- RT 28 phase change material, manufactured by Rubiterm Gmbh, Berlin, Germany;
- Water dispersion of vinyl acetate and alkyl acrylates, produced by Synthos, Oświęcim, Poland;
- Warbonrecycler from the Jastrzębska Coal Company Koks, Jastrzębie-Zdrój, Poland.

#### 2.1.2. Apparatus

- Espec climatic chamber with temperature modulation function;
- COMET MS-6D| MS6R recorder(Comet Electronics, Roznov pod Radhostem, Czech Republic);
- FLIR 7i thermal imaging camera(Flir systems, Meer, Belgium);
- FQA020 C heat flux density sensors (measurement error ± 2%, (Ahlborn, Holzkirchen, Germany);
- PT1000 temperature sensor (measurement error ± 0.3 °C, (Ahlborn, Holzkirchen, Germany);
- MIRA3 scanning microscope by Tescan, Brno, Czech Republic);
- Due X detector (EDS) by Oxford Instrument, Abingdon, Great Britain).

### 2.2. Description of Empirical Research

**Figure 1:**
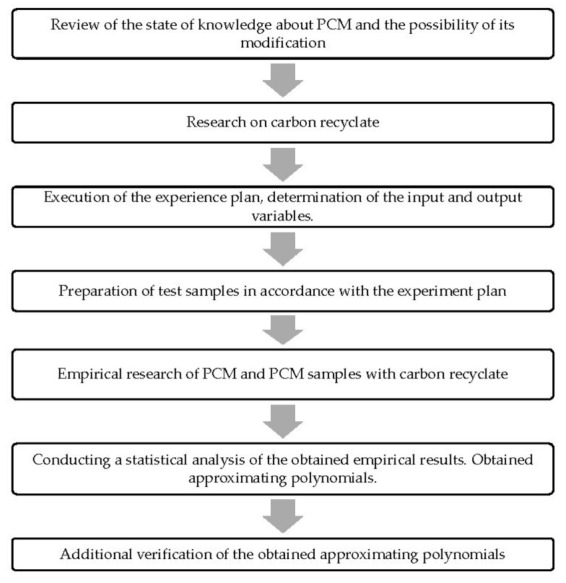
Scheme of the research approach.

In order to increase the clarity of the article, Figure 1 presents a graphical diagram of the research approach.

#### 2.2.1. Properties and Application of Recycled Material

Work on the nature of the obtained pyrolysis products, carried out since the last century, is aimed at the possibility of using them in various sectors of the economy. It is assumed that this process produces 20.9–60% of pyrolytic oil.

From solid products in the narrow range, we obtain 22–48.5% of biochar and with typical values of 38–40%. Gaseous products range from 3–27% [43]. The biochar is solid pyrolysis residue, containing approximately80% elemental carbon [44]. The high-carbon biochar is coke. It is created as a result of high-temperature degassing of coking coal in a set of coking chambers, diaphragm-heated without air access [45,46]. The content of elemental carbon in coke is high—it can even exceed 95%—which is the result of removing gases, liquids, and other pollutants from the coal during pyrolysis. Coke is used primarily in smelting iron in metallurgy, where it is a fuel, a reducing agent, and acts as a “scaffolding”, allowing gas permeability of the charge. It is also used as a high-quality fuel for firing heating boilers, for smelting the non-ferrous metals Zn, Pb, Cu, and for the production of ferroalloys (Cr, Fe + Si, Mn). Research on the physical and chemical properties and combustion of semi-coke and solid pyrolysis products are the subject of current research [47,48]. Due to their technological features and nature, we can distinguish with in coke: blast furnace coke, metallurgical coke, foundry coke, and heating coke.

**Figure 2:**
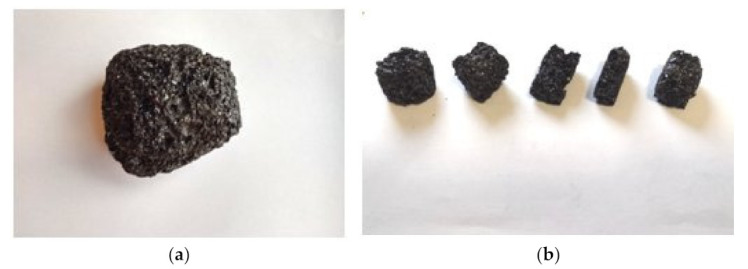
(**a**) Raw recyclate; (**b**) Preparation of experimental samples.

The recyclate used in the research was characterized by a “foam” structure and brittleness (Figure 2). These features indicate that it was formed from carbons with a lower degree of carbonation, where excessive gas was released during carbonization and late shrinkage. The influence of the degree of carbonization on the process of coke formation is closely related to the molecular structure of carbon [49]. As the degree of carbonization increases, the order of aromatic lamellae and their size increase [50].

#### 2.2.2. SEM Research Methodology

Surface morphology and imaging of the waste structure were performed using the Tescan MIRA3 scanning microscope (Tescan, Brno, Czech Republic). In order to determine chemical composition, a field emission and X-ray detector (EDS) from Oxford Instruments was used. The research preparation required spraying the samples with a layer of gold about 30–45 nm thick. This process was carried out in a vacuum sputtering machine Surface morphology and imaging of the waste structure were performed using the Tescan MIRA3 scanning microscope (Tescan, Brno, Czech Republic). In order to determine chemical composition, a field emission and X-ray detector (EDS) from Oxford Instruments was used. The research preparation required spraying the samples with a layer of gold about 30–45 nm thick. This process was carried out in a vacuum sputtering machine. The samples were imaged at four magnifications of 2000, 5000, 20,000, and 50,000 times. The electron accelerating voltage was selected in the range from 10 to 20 kV. The mapping of elements was performed at a magnification of 1000 times. The average time of one mapping was approximately 10 min. X-ray detection covered the energy range from 0 to 10 keV. The surface distribution of elements was performed with the image resolution of 1024 × 1024 pixels. The time of counting the signal to the spectrum from one pixel is 500 microseconds. The elemental composition is the average value of the entire map. The material obtained from carbon recycling used in the research is presented in Figure 2.

The samples were imaged at four magnifications of 2000, 5000, 20,000, and 50,000×. The electron accelerating voltage was selected in the range from 10 to 20 kV. The mapping of elements was performed at a magnification of 1000 times. The average time of one mapping was approximately 10 min. X-ray detection covered the energy range from 0 to 10 keV. The surface distribution of elements was performed with the image resolution of 1024 × 1024 pixels. The time of counting the signal to the spectrum from one pixel is 500 microseconds. The elemental composition is the average value of the entire map. The material obtained from carbon recycling used in the research is presented in Figure 2.

### 2.3. Description of the Statistical Analysis

One of the objectives of this article is to determine the parameters that affect the efficiency of heat distribution within the new PCM composite with carbon recyclate. In addition, determination of the significance level of each variable and their correlation as well as obtaining a mathematical relationship that allows the prediction of the impact of individual input parameters on the efficiency of heat distribution within the PCM package with carbon recyclate was investigated. According to the works [2,4,5,8,37,38,39,41], a significant impact on the efficiency of heat distribution within packages with phase change material are:

- Heating intensity of the PCM package;
- Initial temperature of the PCM package;
- Shape and dimensions of the PCM package.

In turn, the result of the analysis was the heat distribution coefficient of the heat storage package, which represents the difference between the empirical and theoretical amount of heat needed to melt the PCM within the package.

The planning of the experiments necessary to perform and the subsequent analysis of the empirical results and their statistical analyses were carried out in Statistica, version 12. Among the industrial statistics available in the program, the experiment planning (DOE) function was used, which allowed the generation of an incomplete experiment plan with three inputs, one output, and two test repetitions.

The specification of the input quantities and the output quantity of the experiment plan, along with those adopted on the basis of calibration experiments and the physical parameters of PCM and carbon recyclate, are presented in Table 1.

Determination of the relationship between the input quantities and the value of the output quantity was obtained through the use of an incomplete central compositional experiment plan, in accordance with [42]. The calculations related to the incomplete design of the experiment and the statistical analysis of the empirical results were carried out in the Statistica 12 program. The solution of the relationship between the input quantities and the output quantity was to determine the coefficients of the approximating polynomial variables, according to Equation (1):

(1) $$z\;=\;b_{0}\;+\;b_{1}x_{1}\;+\;\cdots \;+\;b_{k}\cdots x_{k}\;+\;b_{12}x_{k}\cdots x_{1}x_{2}\;+\;b_{13}x_{1}x_{3}\;+\;\cdots \;+\;b_{k\;-\;1}x_{k\;-\;1}\;+\;b_{11}x_{1}^{2}\;+\;\cdots \;+\;b_{k}x_{k}^{2}$$

In turn, the required number of experiments was determined on the basis of Equation (2):

(2) $$n\;=\;n_{k}\;+\;n_{\alpha }\;+\;n_{0}$$

where:

$n_{k}$ is thenumber of systems in the plan core

$n_{\alpha }$ is thenumber of systems of the stellar points

$n_{0}$ is thenumber of repetitions of the experiments

**Figure 3:**
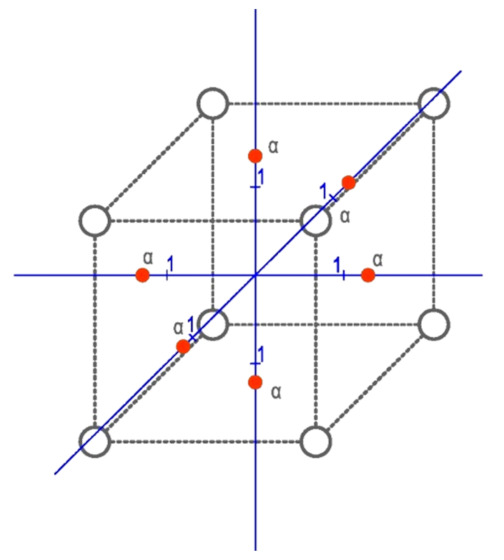
Graphical diagram of the stellar ray of an incomplete, polyselective, orthogonal plan experiment plan.

In order to become independent from the physical interpretations of empirical data analyses, it is necessary to normalize the input values to the <−α, α> interval, where α is the stellar ray. The value of α for the central compositional plan 3/1/15 is, according to [42], α = 1.215. The length of the stellar ray, as shown in Figure 3, was determined as Equation (3).

(3) $$\alpha \;=\;\alpha_{\mathrm{ort}}\;=\;\sqrt{0.5\;[\sqrt{2^{i\;-\;p}\left(2^{i\;-\;p}\;+\;2i\;+\;n_{0}\right)\;-\;2^{i\;-\;p}}}$$

Graphic interpretation of the stellar ray is shown in Figure 3.

The input quantity normalization process was performed according to Equation (4).

(4) $${\overset{˘}{x}}_{k}\;=\;\frac{2\alpha \left(x_{k}\;-\;x_{k.\min}\right)}{x_{k.\max}\;-\;x_{k.\min}}\;-\;\alpha$$

Then, after performing the necessary experiments, in order to obtain the actual value of the output quantity, the inverse operation was performed in accordance with Equation (5)

(5) $$x_{k}\;=\;\left(\frac{{\overset{ˇ}{x}}_{k}\;+\;\alpha }{2\alpha }\right)\;\cdot \;\left(x_{k.\max}\;-\;x_{k.\min}\right)\;+\;x_{k.\min}$$

The layout of the experiment plan is a set of tested input variables for which the output values are obtained, according to relationship (6)

(6) $$\left\{x_{1/u},x_{2/u}\ldots x_{k/u},x_{i/u}\right\}$$

Then, an adequacy analysis of the obtained model was carried out. Therefore, a substantive analysis was carried out in terms of:

- Assumptions of the proper form of the approximating equation;
- Possible omission of a quantity important for the object’s function;
- Correctness of the adopted range of input variables.

The ranges of input variability included in the experiment plan performed in the STATISTICA 12 program are presented in Table 2.

**Figure 4:**
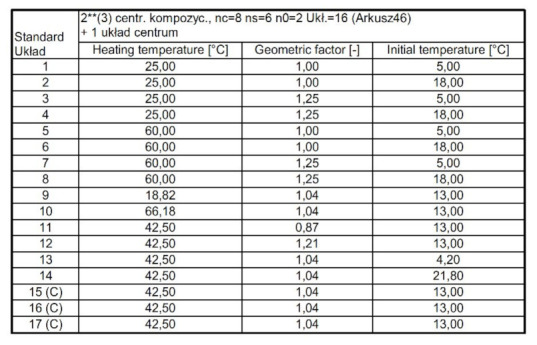
List of experiments necessary to perform, according to the incomplete experiment plan of the Statistica program.

**Figure 5:**
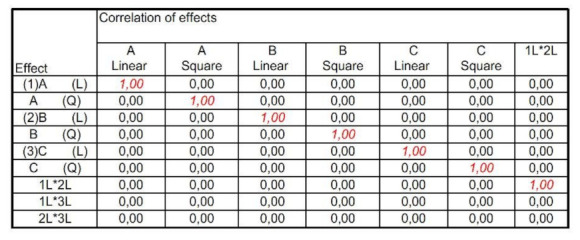
Summary of the effects of input variables and their correlations analyzed in the experiment plan in the Statistica program.

The experiments were executed in accordance with the plan. The lists necessary for the implementation of the experiments and the correlation of individual effects considered in the analysis are presented in Figure 4 and Figure 5.

#### 2.3.1. Research Position

**Figure 6:**
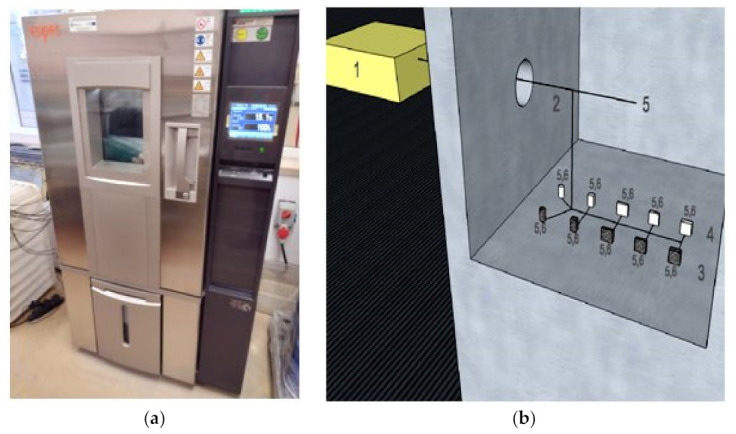
(**a**) A photograph of the Espec climatic chamber during the research; (**b**) Scheme of the test stand, where: 1, recorder; 2, interior of the climate chamber; 3, research samples of the PCM composite with recyclate; 4, pure PCM test samples; 5, temperature sensors; 6, heat flux density sensors.

The experimental studies were carried out in an Espec climatic chamber with the functions of temperature and humidity modulation, in accordance with the assumptions of the incomplete central compositional plan of the experiment presented in Figure 4. During the experiments, samples with pure phase change material, as well as samples with PCM and steel plant recyclate, were tested in parallel with identical geometry, as shown in Table 2. During each experiment, the samples were brought to the initial temperature, then heated and cooled to the desired temperature, in accordance with the conditions set in the experiment plan included in Figure 4. The process of stabilizing the temperature of the samples lasted 3 h, heating up for 3 h and cooling down for 15 h. The heating temperature of the samples during subsequent experiments was determined on the basis of the experiment plan according to Figure 4. During the tests, air temperature values, sample temperature, and the density of the heat flux flowing through the samples were recorded. The scheme of the test stand with the location of the sensors is presented in Figure 6.

#### 2.3.2. Preparation of Experimental Samples

**Figure 7:**
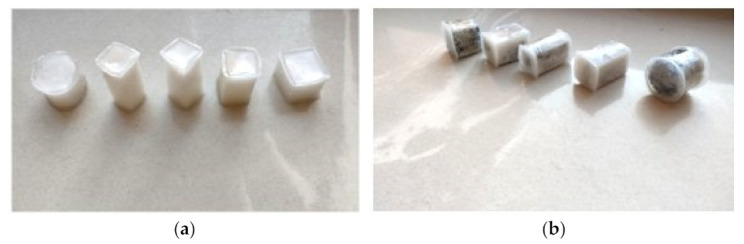
(**a**) Experimental samples containing pure PCM; (**b**) test samples containing PCM with carbon recyclate.

The research samples of the heat accumulators were made of RT 28 phase change material and the copolymer coating of vinyl acetate and alkyl acrylates obtained from water dispersion. Tight coatings of the samples were obtained by immersing the solid PCM in an aqueous dispersion of the copolymer. This was possible due to the lower glass transition temperature of the dispersion than the melting point of PCM. The shape and geometry of the samples as the shape factor Gf were selected in accordance with the assumptions of the central compositional experiment plan according to [42], in accordance with Figure 4. Samples containing PCM and the material were recycled by cutting them out according to the geometry of carbon recyclate set in the experiment plan. The cut components were then immersed in liquid PCM for a period of 12 h to fill the pores of the PCM samples. Then, after cooling and solidifying the PCM, the test samples were immersed in an aqueous dispersion of a copolymer of vinyl acetate and alkyl acrylates, in the same way as the samples with pure PCM. Prior to the commencement of the actual tests, leakage tests of the produced samples were carried out. Figure 7 presents the process of preparing experimental samples.

Prepared samples of PCM packets and PCM composite packets with carbon recyclate were subjected to cyclic heating and cooling tests according to the experiment plan to experimentally determine the ratio between the theoretical (derived from calculations) and the actual (empirically derived) amount of heat needed to completely melt the PCM in the samples. The quotient between the obtained from the tests and the calculated amount of heat needed to melt samples of different geometries is the heat distribution coefficient, which is also the output value of the test plan response function. The above differences in the amounts of heat absorbed by melting were determined according to the Equations (7)–(9).

(7) $$\eta_{D}\;=\;\frac{Q_{E}}{Q_{T}}$$

where: η_Q_ is the efficiency of heat distribution; Q_E_ is the actual amount of absorbed heat; Q_T_ is the calculated amount of absorbed heat.

(8) $$Q_{T}\;=\;\left\{\begin{array}{c} \begin{array}{ccc} {\mathrm{if}\;T}_{P}\;<\;T_{l} & \to & m_{s}\;\cdot \;C_{w.s}\int_{t\;=\;1}^{t\;=\;O}∆T_{T}\mathrm{dt} \end{array}\\ \begin{array}{ccc} {\mathrm{if}\;T}_{P}\;\geq \;T_{l} & \to & m_{l}\int_{t\;=\;O}^{t\;=\;n}∆H_{T}\mathrm{dt} \end{array} \end{array}\right.$$

where: T_p_ is the sample temperature; T_l_ is the melting point; ms is the mass of solid PCM; C_w.s_ is the specific heat of solid PCM; ΔT_E_ is the theoretical temperature increase of PCM samples; m_l_ is the mass of molten PCM; ΔH_T_ is the PCM melting/solidification enthalpy value.

(9) $$Q_{E}=\left\{\begin{array}{c} \begin{array}{ccc} {\mathrm{if}\;T}_{P}\;<\;T_{l} & \to & A_{p}\int_{t\;=\;1}^{t\;=\;O}q_{\mathrm{rej}}\mathrm{dt} \end{array}\\ \begin{array}{ccc} {\mathrm{if}\;T}_{P}\;\geq \;T_{l} & \to & m_{l}\int_{t\;=\;O}^{t\;=\;n}∆H_{E.\mathrm{rej}}\mathrm{dt} \end{array} \end{array}\right.$$

where: T_p_ is the sample temperature; T_l_ is the melting point; A_P_ is the side surface of the PCM package; q_rej_ is the heat flux density passing through the PCM package; m_l_ is the mass of molten PCM; ΔH_E.rej_ is the value of the melting/solidification enthalpy of PCM.

#### 2.3.3. Model Adequacy Assessment

In order to additionally verify the obtained response functions, a statistical test was performed to confirm the adequacy of the adopted form of the approximating function.

The adequacy of the adopted model was verified by the Fisher–Snedecor test together with the calculation of the approximation error with the selected function f(x). Then, the values of the error function were compared with the randomly determined ad value. Obtaining an a_d_ value greater than the additionally calculated error amount a_dmax_ makes the model adequate. The methods of determining the function approximation error most frequently quoted in the literature [5,7,40,42] are:

- Maximum absolute approximation error;
- Maximum relative error;
- Mean square error.

The Fisher–Snedecor statistical test was carried out by verifying that the conditions of the null hypothesis and the alternative hypothesis were met in accordance with Equation (10).

(10) $$H:\;\sigma_{a}^{2}\;\Leftrightarrow \;\sigma^{2}$$

Null hypothesis H0: $\sigma_{0}^{2}\;=\;\sigma^{2}$ meaning the adequate model.

Alternative hypothesis H1: $\sigma_{a}^{2}\;>\;\sigma^{2}$ denoting an inadequate model.

In turn, the statistical verification of the experimental design was performed by comparing the calculated value of the test function, in accordance with the formula, with the permissible critical value, in accordance with Equations (11) and (12).

(11) $$F\;\leq \;F_{\alpha ,f_{1},f_{2}}$$

(12) $$F\;=\;\frac{\sigma_{a}^{2}}{\sigma^{2}}$$

where:

$\sigma_{a}^{2}$ is the adequacy variance, which is calculated from the Equation (13).

(13) $$\sigma_{a}^{2}\;=\;\frac{1}{f_{1}}\overset{n}{\underset{u\;=\;1}{\sum }}{\left({\bar{x}}_{u}\;-\;{\hat{x}}_{u}\right)}^{2}$$

$\sigma^{2}$ is variance of the inaccuracy of empirical measurements, determined from the Equation (14).

(14) $$\sigma^{2}\;=\;\frac{1}{f_{2}}\overset{m}{\underset{u\;=\;1}{\sum }}{\left({\bar{y}}_{u}\;-\;{\hat{y}}_{u}\right)}^{2}$$

where:

$f_{1}$ = (n − 1) number of degrees of freedom of variance $\;\sigma_{a}^{2}$

$f_{2}$ = (m − 1) number of degrees of freedom of the variance $\;\sigma^{2}$

α = 0.05 significance level

## 3. Results

### 3.1. SEM/EDS Analysis Results

**Figure 8:**
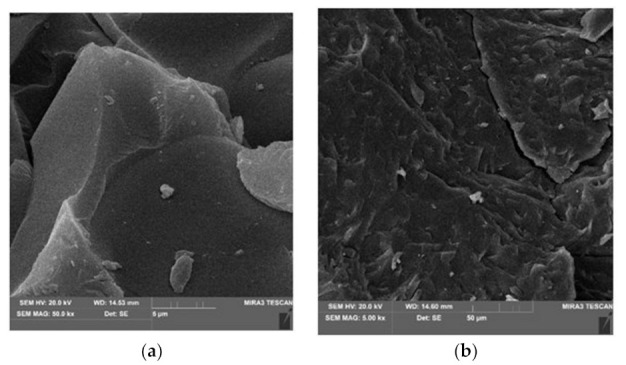

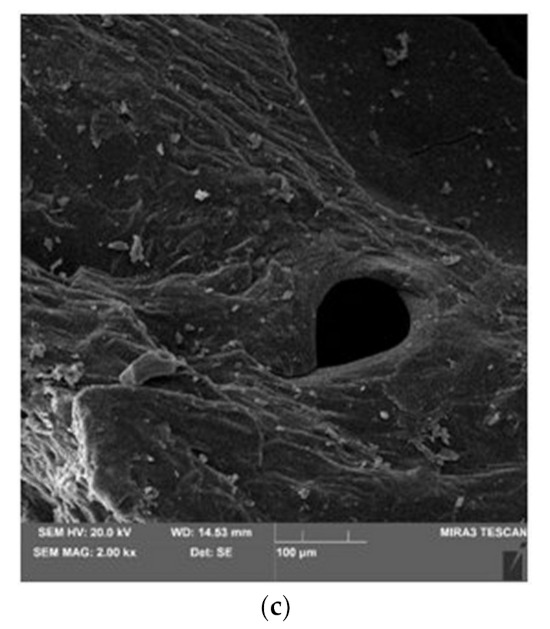
The nature of the microstructural surfaces of the waste material. SEM image: (**a**) semicircular shell fracture, (**b**) surfaces with a spiked, uneven fracture, (**c**) oval, unfilled pores.

The analysis of the microstructural surfaces of the waste material using SEM showed the massive nature of their texture. There were smoothed fragments with a semicircular shell fracture (Figure 8a) and surfaces with a spiked, uneven fracture (Figure 8b). Oval, unfilled pores are visible in places (Figure 8c).

**Figure 9:**
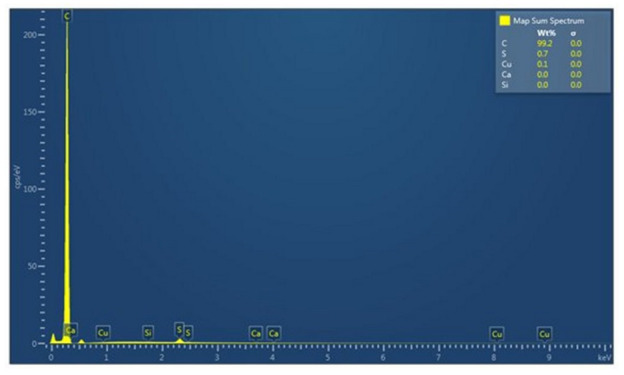
Chemical analysis from the point of analyzed waste. EDS.

The conducted chemical analyses of EDS show slight differences in the chemical composition of the analyzed waste (Figure 9).

**Figure 10:**
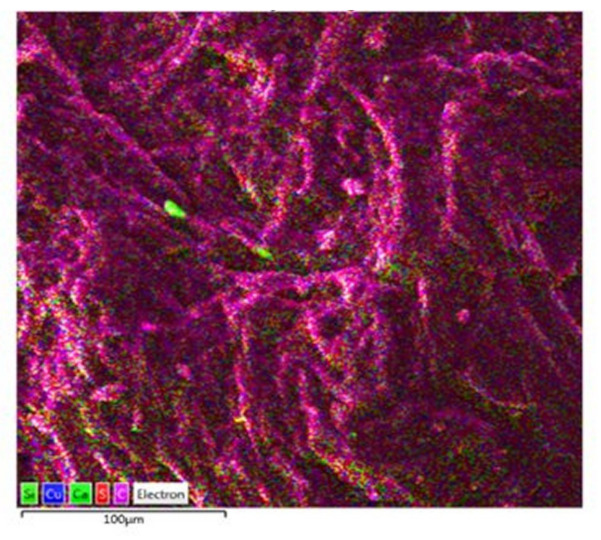
Surface distribution of the identified elements.

**Figure 11:**
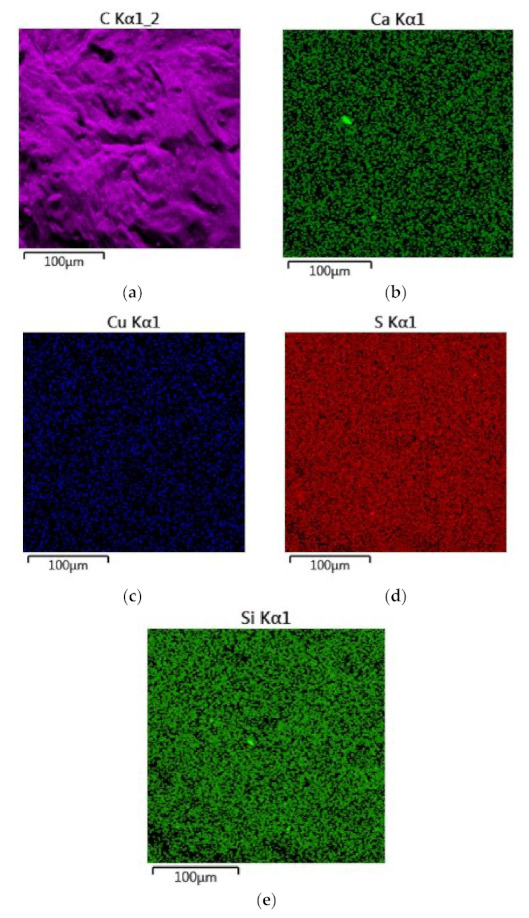
Distribution of individual elements identified on the microstructural surface; (**a**) carbon; (**b**) calcium; (**c**) cupper; (**d**) sulfur; (**e**) silicon.

Analysis in the micro-area showed that the main component of the waste is carbon, which homogeneously covers the microstructural surface (Figure 10 and Figure 11a). Moreover, in the analyzed material, insignificant amounts of sulfur (0.66 wt.%) and copper (0.1 wt.%) were found (Figure 9 and Figure 11d,c). In trace amounts at the level of 0.02 wt.%, silica and calcium can be identified (Figure 9 and Figure 11e,b). The figure shows the surface distribution of the elements found in the tested material (Figure 11).

At the current stage of the research, we can confirm that the recyclant used in the experimental research is of a carbon nature. In the case of chemical analysis of the ingredients, the sulfur content of 0.66 wt.% deserves attention. Increased sulfur content makes the resulting metal “brittle when hot” [46]. The sulfur present in coke has a detrimental effect on the natural environment. It also causes faster corrosion of various devices and contaminates catalysts. The content of total sulfur in coke produced from Polish coal is usually 0.7–1.1% [51]. Due to the nature of the tested materials, a detailed analysis of the chemical composition and trace elements requires the extension of the research methodology. This will be presented in the next research stage.

### 3.2. The Results of Empirical Research

**Figure 12:**
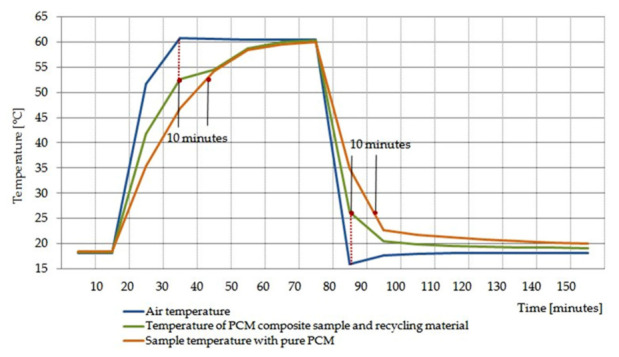
Chart of recorded temperature changes of pure PCM packets and packets with PCM composite with carbon recyclate during their heating and cooling according to the assumptions of experiment No. 6.

The obtained results of empirical research conducted according to the assumptions of the experimental plans proved the improvement of heat distribution within the heat accumulator in the form of a PCM composite with carbon recyclate compared to the reference heat accumulator with pure PCM. The comparison of the thermal performance of the packets of PCM composites with packets of pure PCM, an example of cubic samples, made it possible to observe a reduction of the heating time of PCM during its melting and a reduction of the cooling time during solidification of about 10 min. The shortening of the heating and cooling time of the PCM composite with carbon recyclate, compared to pure PCM, with similar PCM content in each sample proves the improvement of the efficiency of stored heat distribution. A graphic presentation of the temperature distribution of both batteries tested in parallel is presented in Figure 12.

**Figure 13:**
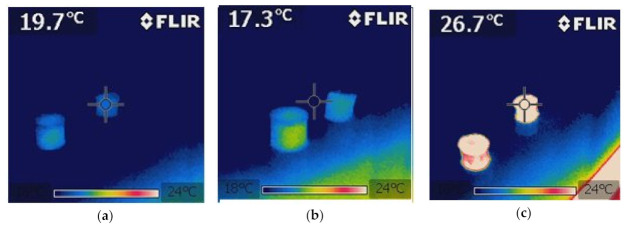

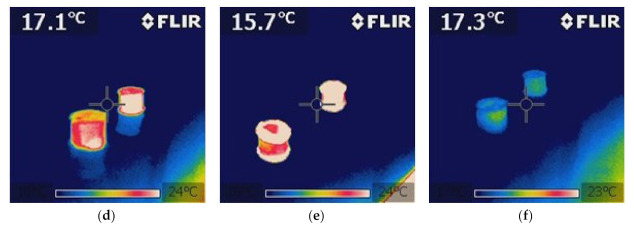
Thermograms of heating and cooling samples with pure PCM and with composite PCM and coke recyclate. (**a**) steady state before heating, (**b**) initial heating phase, (**c**) steady state after sample heating, (**d**) start of sample cooling, (**e**) start of sample cooling, (**f**) the final stage of cooling the samples.

In order to additionally verify the results of the empirical temperature distribution of the samples of the PCM composite with carbon recyclate and pure PCM in qualitative terms, the Flir 7i thermal imaging camera was used for heated and cooled cylindrical samples. Thermograms made with the thermal imaging camera of the tested samples are shown in Figure 13. In Figure 13b, faster and more intense heating of the PCM composite can be noticed compared to pure PCM. On the other hand, during cooling of heated samples containing liquid phase change material, faster cooling was noticed in samples with PCM composite and carbon recyclate compared to pure PCM, Figure 13e.

### 3.3. Statistical Analysis Results

**Figure 14:**
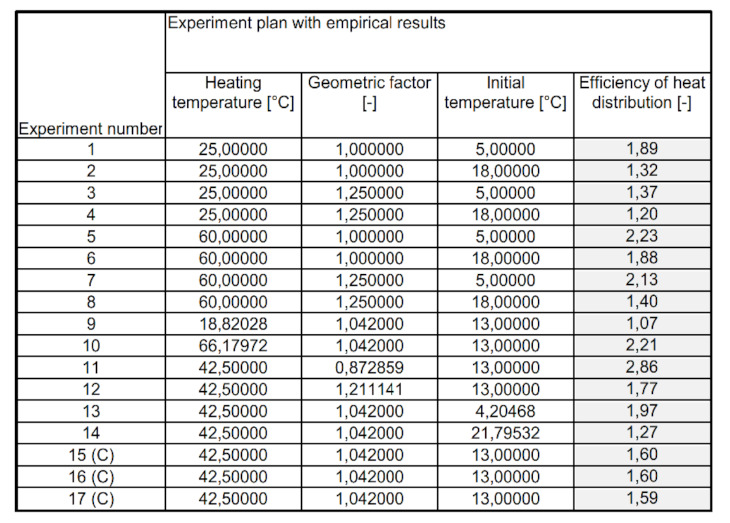
List of empirically determined heat distribution coefficients for the PCM composite with coke recyclate.

The obtained results of the research carried out in accordance with Figure 4 allowed for the implementation of the statistical analysis. It was carried out on the basis of two combinations: checking the approximating function of the output variable was performed for the significance level α = 0.05, and the Pareto analysis of individual effects includes arranging the input quantity in the order of significance on the output variable. The obtained empirical results of heat distribution coefficients for the PCM composite with coke recyclate, for the set experiments, are determined according to Equation (7) and presented in Figure 14.

**Figure 15:**
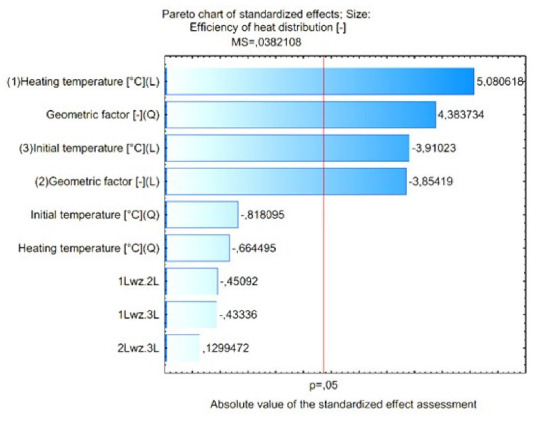
Pareto diagram of the influence of the analyzed input quantities and their correlation on the value of the output expression.

**Figure 16:**
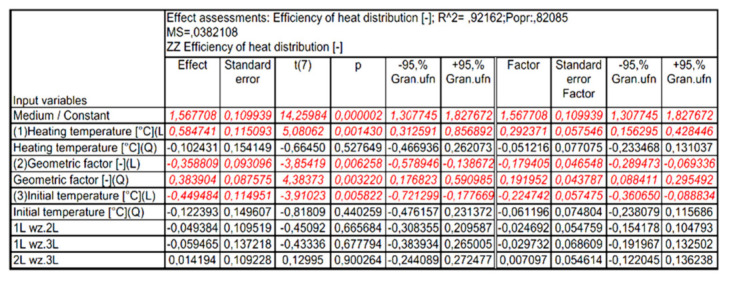
Summary of independent variable assessments on the baseline quantity.

**Figure 17:**
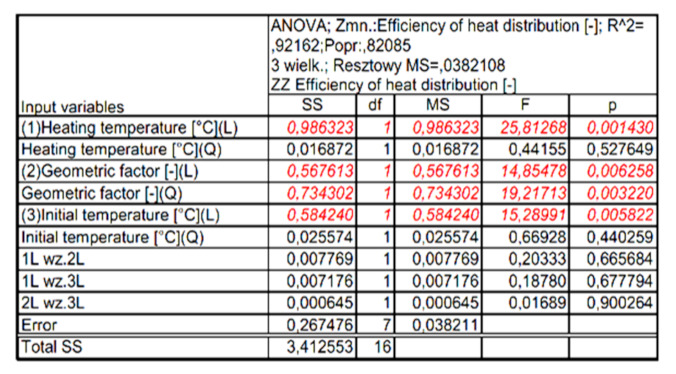
Summary of the results of ANOVA analysis of input variables on the value of the output variable, where: df, degrees of freedom; MS, pure error; SS, variance; F, statistic value, coefficient informing about the contribution of a given variable to the prediction; p, significance level, coefficient correlation; R^2^, regression coefficient.

Figure 15 shows the Pareto diagram of the influence of the analyzed input quantities and their correlation with the value of the output expression. The results in Figure 15 show that for the assumed 5% error level, these values are significant: heating temperature (L), geometric factor (Q), initial temperature (L) and geometric factor (L). Detailed results of the analysis performed are presented in Figure 16 and Figure 17.

**Figure 18:**
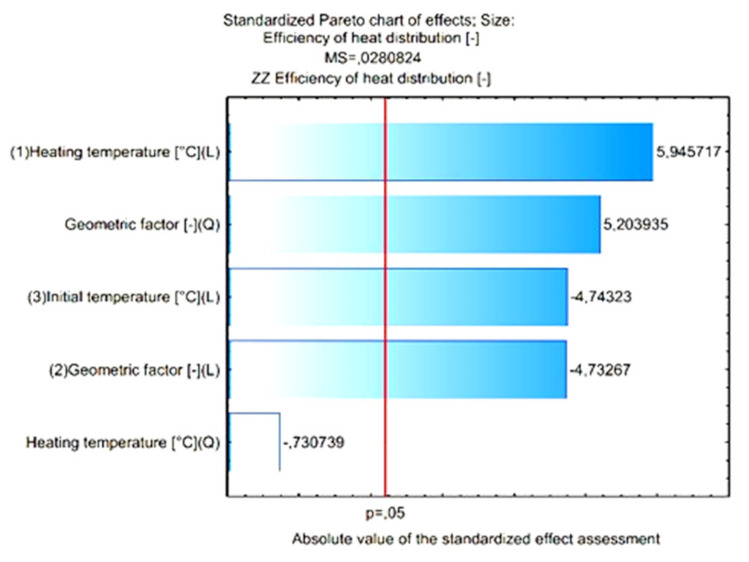
Pareto diagram of the influence of the analyzed input quantities and their correlationswith the value of the output expression, excluding insignificant quantities.

**Figure 19:**
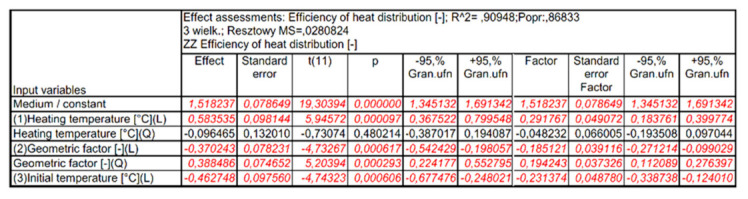
Summary of independent variables assessments on the output quantity, excluding insignificant quantities.

**Figure 20:**
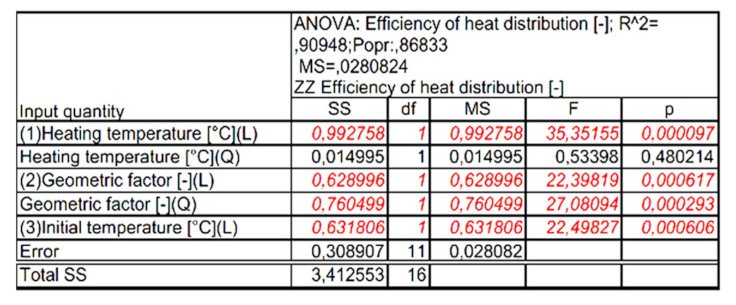
Summary of the results of the ANOVA analysis of the input variables on the value of the output variable, excluding insignificant quantities.

In the next stage of the analysis, statistically insignificant input variables and their correlations, which—according to Figure 15—are characterized by a significance level below α = 0.05, were included in the error component of the analysis. After including statistically insignificant words in the analysis error component, no decrease in the number of input quantities influencing the values of the output quantity was observed. The new graph of the Pareto analysis and the assessment of the effects of the analyzed variables, after excluding the irrelevant components, are presented in Figure 18, Figure 19 and Figure 20.

Finally, the four components that are statistically significant in determining the output quantity (heat distribution efficiency) are: heating temperature (L), geometric factor (L), geometric factor (Q), and initial temperature (Q).

**Figure 21:**
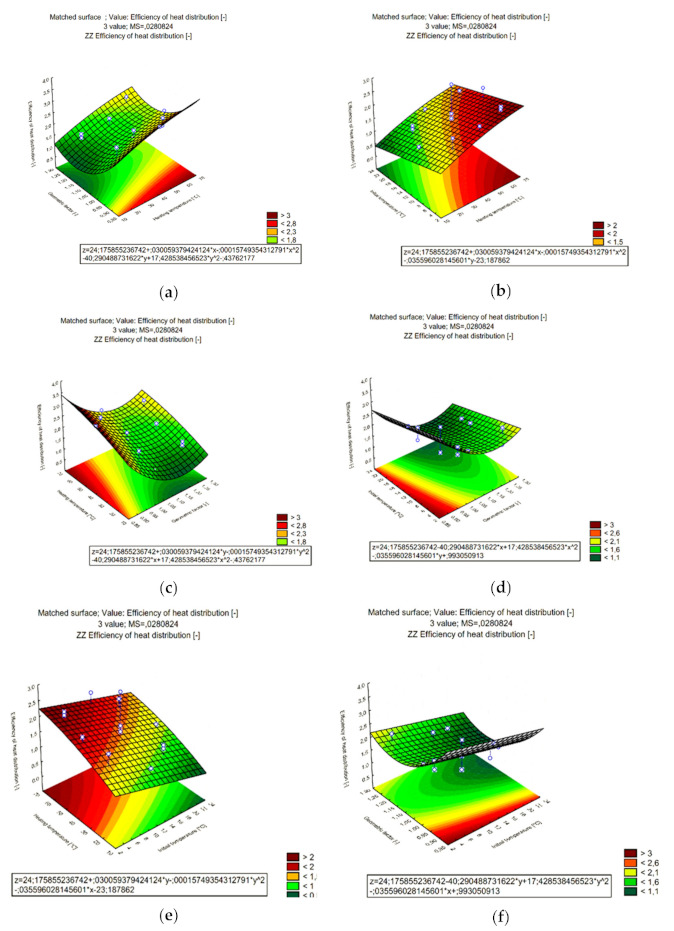
Formation of graphs of approximating functions in the form of planes of input variable responses to the value of the heat distribution coefficient of the PCM composite with carbon recyclate, (**a**) The plane of the heating temperature response to the geometrical coefficient, (**b**) The plane of the heating temperature response to the initiation temperature, (**c**) The plane of geometrical coefficient response to the heating temperature, (**d**) The plane of geometrical coefficient response to the initiation temperature, (**e**) The plane of the initiation temperature response to the heating temperature, (**f**) The plane of the initiation temperature response to geometrical coefficient.

The results of the experiments carried out in accordance with the experiment plan and the statistical analysis of the obtained empirical results are functions of the approximating polynomials, presented in Table 3. The graphical form of the approximating functions in the form of response planes is presented in Figure 21.

The approximating function equations presented in Table 3 were obtained as part of the statistical analysis of empirical results. The obtained equations can be used in future works in the assumed domain of individual variables included in Table 2.

### 3.4. Verification of the Obtained Approximating Functions

In order to verify the obtained functions of the object in the form of the approximating polynomial, the null hypothesis condition was checked according to Equations (10)–(14) of the Fisher–Snedecor test. The test was carried out by comparing the variance values of the separated empirical results obtained during the research and the variance of the values calculated in accordance with the approximating function formulas presented in Table 3.The analysis of the matching of empirical to theoretical values was performed using the quasi-Newton method, as in the works [5,7,37]. The determination of the minimum of the error function was performed in accordance with Equation (15).

(15) $$\min y\;=\;\overset{n}{\underset{t\;=\;1}{\sum }}{\left[\eta_{D.E}\;-\;\eta_{D.T}\right]}^{2}$$

In turn, the value of the determination coefficient was obtained thanks to dependence (16).

(16) $$R^{2}\;=\;1\;-\;\frac{\sum_{t\;=\;1}^{n}{\left({\hat{y}}_{t}\;-\;\bar{y}\right)}^{2}}{\sum_{t\;=\;1}^{n}{\left(y_{t}\;-\;\bar{y}\right)}^{2}}$$

where: *y_t_* is the actual value of the variable; ${\hat{y}}_{t}$ is the theoretical value of the explanatory variable; $\bar{y}$ is the arithmetic mean of the independent variable.

The analysis of the matching of empirical to theoretical results is justified, as it allows for additional verification of the results of the Fisher–Snedecor test, which is sensitive to the point dispersion of the analyzed measurements.

**Figure 22:**
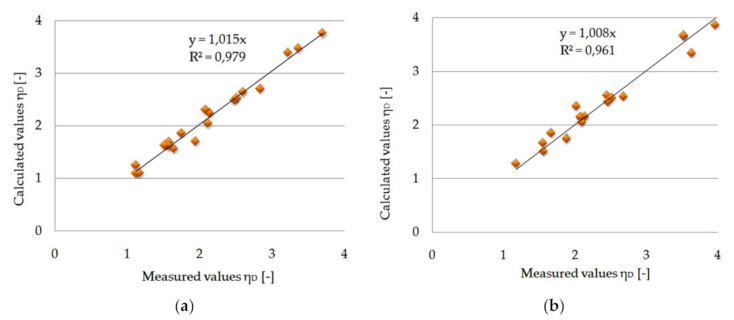
(**a**) The results of the test for fitting the theoretical quantities to the empirical heat distribution coefficient for pure PCM packages (**b**) The results of the test of fitting the theoretical values to the empirical heat distribution coefficient for packages with PCM and carbon recyclate.

Graphical adjustment of empirical values to the theoretical heat distribution coefficient, the values of directional coefficients and determination coefficients, and test results for packages with pure PCM and for the composite PCM with carbon recyclate are presented in Figure 22.

The results of matching randomly selected data, separated from the data considered in the statistical analysis presented in Figure 22, with the results calculated with the use of approximating functions prove their high matching. This fact is supported by the uniform distribution of points on the diagonals of Figure 22 and the values of the “a” directional coefficients and R^2^ determination coefficients, which are close to unity.

## 4. Discussion

The conducted experimental studies and their subsequent statistical and substantive analysis proved the legitimacy of the use of carbon recyclate, a material used to improve the distribution of heat stored within organic material.

The analysis of the structural–textural surface shows a homogeneous distribution of carbon in the matrix of the recycled material used. This property has a positive effect on the composite nature of the phase change material. The chemical analyses carried out in the micro-area show that the carbon content is very high, amounting to over 90%, and is in line with the assumption of the carbon recyclate. In connection with the EU directives, anti-smog resolutions and the prohibition of the use of solid fuels, coal, wood, and heating coke, the subject of the article in the aspect of the management of carbon recyclate as a composite phase change material is part of the currently created circular economy.

The improvement in the efficiency of heat distribution within the tested composite, as compared to the reference pure PCM, was characterized by a reduction of its heating and cooling time by 10 min in the initial stages of both processes.

During the conducted research, no chemical reactions were observed between the carbon recyclate and the PCM, RT28 used for the tests, or the applied copolymer coating of vinyl acetate and alkyl acrylates, which is one of the conditions necessary for the creation of a new composite.

The performed statistical analysis proved the statistical significance of all three assumed input quantities. The differences between the significance level of each of the input variables listed on the value of the output variable do not exceed 20.5%. The obtained model is a useful tool supporting the design of future composite PCM heat accumulators.

The current limitation of the use of the new PCM composite and carbon recyclateis the dimensions of heat storage packages (should not be larger than 30 mm). Additionally, the application of the new composite is limited by the maximum temperature value of 70 °C. Above this temperature, the polymer coating may fail. The solution of the abovementioned limitations, as well as the verification of the composite operation in a longer period of thermal operation and on a larger scale, will be implemented in subsequent stages of the research.

The results obtained and presented in the study confirm the legitimacy of creating a new composite based on organic PCM in the form of a mixture of saturated aliphatic hydrocarbons, carbon recyclate, and copolymer coatings of vinyl acetate and alkyl acrylates in order to obtain a more energy-efficient phase change heat accumulator. The results of the work are a response to the current problem of reducing the demand for thermal energy in buildings and elements of heating systems of electric car, which is part of the issues of recycling, renewable energy, low-emission building design and sustainable construction.

The obtained results of experimental studies also indicate a new direction of application of waste mineral resources. The presented proposal for a technological solution for the utilization of waste material in the form of a PCM-based composite is an innovative aspect of the presented work. It proves the legitimacy of using carbon recyclate as a cheaper and easily available replacement for copper, aluminum, and graphite, which will also improve heat conduction through solid PCM.

## Figures and Tables

**Table 1:** Summary of input and output values within the framework of an incomplete experiment plan.

Input Quantity	Output Quantities
Heating temperature [25; 60]	Eficiency of heat distribution [;]
Initial temperature [5; 18]	
Geometric factor [1.00; 1.25]	

**Table 2:** Ranges of variation of the input quantities of the experiment plan.

Input Quantity	Range of Variation of the Input Quantity	Middle of the Range of the Input Variable
Heating temperature [°C]	T_Heat.min_ < T_Heat_ < T_Heat.max_	(25, 42.5, 60)
Initial temperature [°C]	T_Initial.min_ < T_Initial_ < T_Initial.max_	(5, 13, 18)
Geometric factor [–]	G_f.min_ < G_f_ < G_f.max_	(1.00; 1.04; 1.25)

**Table 3:** Summary of the obtained functions approximating the object functions.

Function	Formula
η_D_ = *f* (T_Heating_, G_f_)	24.176 + 0.030 · T_H_ − 1.575 · 10^−3^ · T_H_^2^− 40.290 · G_f_ + 17.429 · G_f_^2^ − 0.438
η_D_ = *f* (T_Heating_, T_Initial_)	24.176 + 0.030 · T_H_ + 1.575 · 10^−3^ · T_H_^2^ − 0.036 · T_I_− 23.189
η_D_ = *f* (G_f_, T_Heating_)	24.176 + 0.030 · G_f_ − 1.575 · 10^−3^ · G_f_^2^ − 40.290 · G_f_ + 17.429 · T_H_^2^ − 0.438
η_D_ = *f* (G_f_, T_Initial_)	24.176 − 40.290 · G_f_ − 17.429 · G_f_^2^− 0.356 · T_I_+ − 0.993
η_D_ = *f* (T_Initial_, T_Heating_)	24.176 + 0.030 · T_I_ + 1.575 · 10^−3^ · T_I_^2^ − 0.036 · T_H_− 23.189
η_D_ = *f* (T_Initial_, G_f_)	24.176 − 40.290 · T_I_ + 17.429 · T_I_^2^ − 0.356 · G_f_+ − 0.993

## Data Availability

Not applicable.

## Nomenclature

**Symbol**	**Name**	**Unit**
df	Degrees of freedom	(-)
SS	Variance	(-)
F	Statistic value	(-)
b*	Coefficient informing about the contribution of a given variable to the prediction	(-)
p	Significance level	(-)
MS	Pure mistake	(-)
R^2^	Coefficient of determination	(-)
f_1_, f_2_	Degrees of freedom	(-)
T_Heat_	Heating temperature	(°C)
T_Initial_	Initial temperature	(°C)
G_f_	Geometric factor	(-)
η_D_	Eficiency of heat dystrybution	(-)
α	The length of the interstellar ray	(-)
$\sigma_{a}^{2}$	Adequacy variance	(-)
$\sigma^{2}$	Variance of inaccuracy	(-)
PCM	Phase change materials	(-)
SEM	Scanning electron microscopy	(-)
EDS	due X energy dispersion microscope	(-)
$n_{k}$	number of systems in the plan core	(-)
$n_{\alpha }$	number of systems of the stellar points	(-)
$n_{0}$	number of repetitions of the experiments	(-)
T_p_	sample temperature	(°C)
T_l_	melting point	(°C)
C_w.s_	specific heat of solid PCM	(J/g · K)
A_P_	side surface of the PCM package	(m_2_)
q_rej_	heat flux density passing through the PCM package	(W/m^2^)
m_l_	mass of molten PCM	(g)
ΔH_E.rej_	value of the melting/solidification enthalpy of PCM.	(J/g)
ΔT_E_	theoretical temperature increase of PCM sample	(°C)
ΔH_T_	PCM melting/solidification enthalpy value	(J/g)
